# Supplementary material for: Intestinal and Extraintestinal Pathotypes of Escherichia coli Are Prevalent in Food Prepared and Marketed on the Streets from the Central Zone of Mexico and Exhibit a Differential Phenotype of Resistance Against Antibiotics
Source: Antibiotics (Basel). 2025 Apr 16;14(4):406. doi: 10.3390/antibiotics14040406 (PMC12024313; doi:10.3390/antibiotics14040406)

Table S1. Food samples studied. The table shows the type of food and abbreviation of the samples obtained in the study areas by year.

Sample	Year of Sampling	Food Type	Description
**G1-21**	2021	Group 1	**Group 1:** Sauces made from vegetables with or without heating procedures.<br><br>**Group 2:** Raw vegetables.<br><br>**Group 3:** Whole fruits, shakes, and juices.<br><br>**Group 4:** Cooked meat in different presentations and tortillas, bread, and other food made with flour that underwent heating procedures.
**G2-21**		Group2	
**G3-21**		Group 3	
**G4-21**		Group 4	
**G1-22**	2022	Group 1	
**G3-22**		Group 3	
**G4-22**		Group 4	
**G1-23**	2023	Group 1	
**G2-23**		Group 2	
**G3-23**		Group 3	
**G4-23**		Group 4	

Figure S1. Growth of bacterial colonies isolated from food samples. (**a**) creamy and fuchsia colonies characteristic of *E. coli,* are observed in Mac Conkey agar; (**b**) green-metallic precipitates that confirm the presence of *E. coli* in EMB agar; (**c**) identification of non-fermentative lactose colonies such as *Salmonella* spp. and *Shigella* ssp. in Mac Conkey agar; (**d**) possible identification of *klebsiella* or *Proteus* in EMB agar.

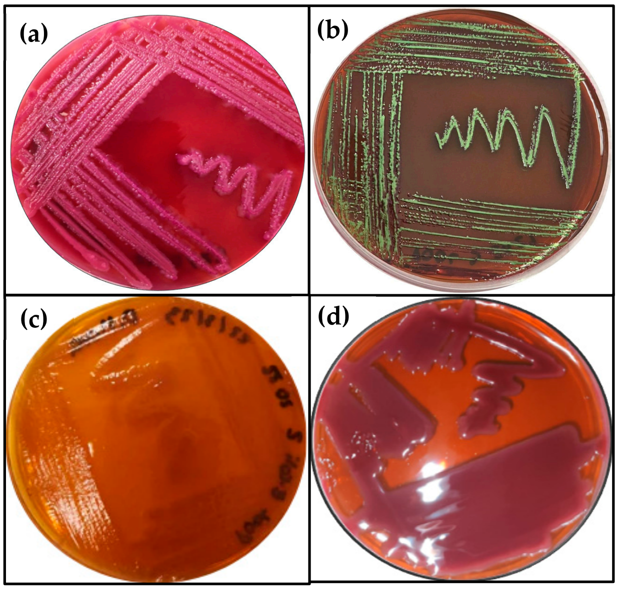

Figure S2. Identification of pathogenic genes in the genome of *E coli* isolated from food. (**a**) Amplicons of the *vat* gene encoding for vacuolating toxin at a size of 289bp; (**b)** Amplicons of the *cnf1* gene encoding for cytotoxic necrotic factor 1, with a size of 498bp; (**c**) Amplicons of the gen *lt*, encode for the thermostable toxin, with a size of 450bp; (**d**) Amplicon of the gen *hlyA* encoding for alpha-hemolysin at a size of 1177bp; (**e**) Amplicon of the gene *afa*, encodes for the afimbrial adhesins, with a size of 809bp.

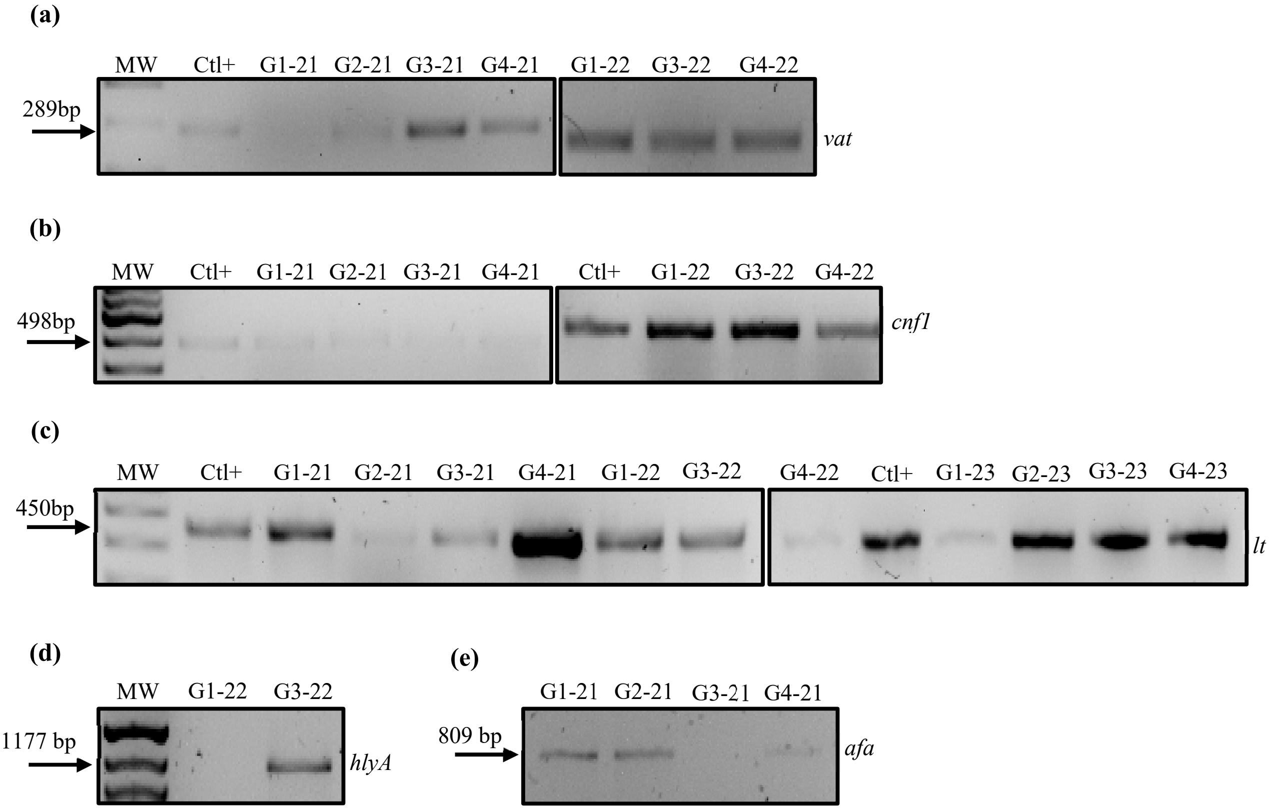

Figure S3. Identification of the antibiotic resistance genes in the genome of *E coli* isolated from food. (**a**) Amplicons of the gen *floR*, encode for a chloramphenicol efflux pump, with a size of 398bp; (**b**) Amplicons of the gene *strA*, encodes for aminoglycoside phosphotransferase, with a size of 548bp; (**c**) Amplicons of the gen *sul1*, encoding for sulfonamide resistant dihydropteroate synthase, with a size of 698bp; (**d**) Amplicon of the gen *tetA*, encodes for major facilitator superfamily efflux pump, with a size of 210bp; (**e**) Amplicon of the gene *catA1*, encode for a chloramphenicol acetyltransferase, with a size of 547bp; (**f**) Amplicons of the gene *qnrS* encoding for quinolone resistance protein, with a size of 428bp.

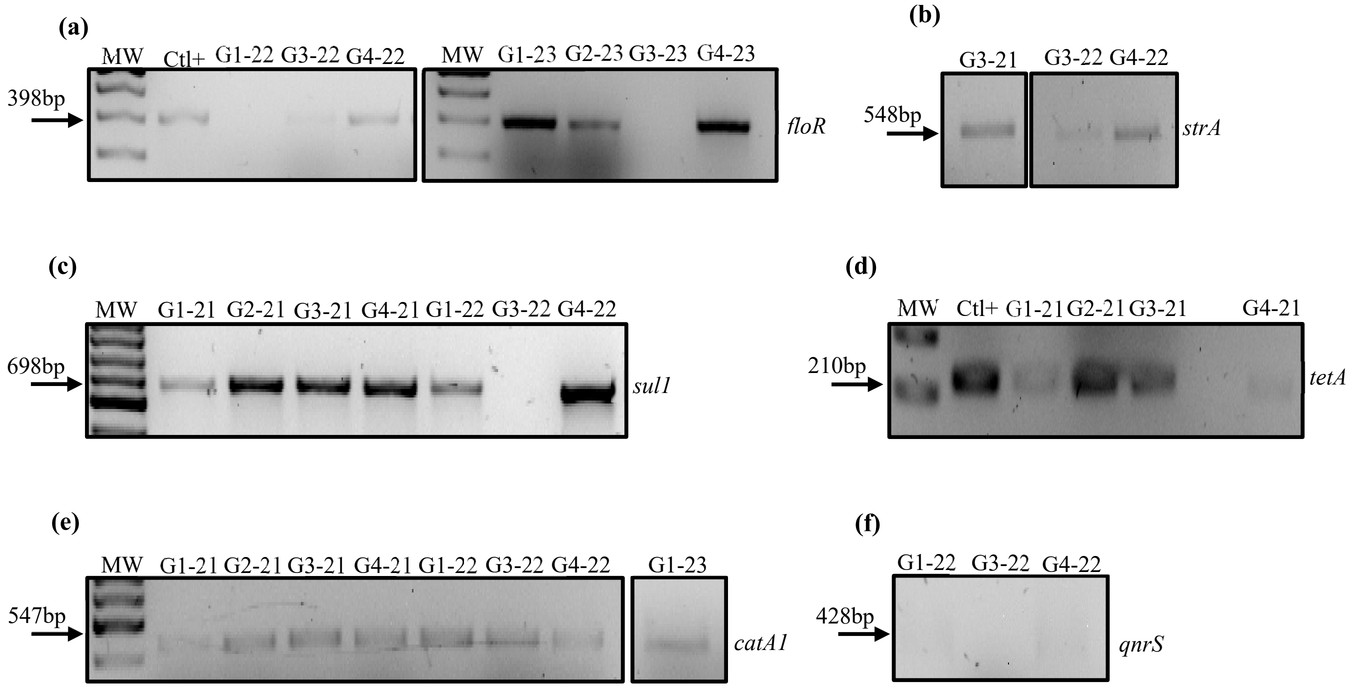

Table S2. Primers sequences of pathogenic genes.

E. coli pathotype	Locus	Primers	Amplicon (bp)	Reference
ETEC	*lt*	F:5′-GGC GAC AGA TTA TAC CGT GC-3′	450	[57]
		R:5′-CGG TCT CTA TAT TCC CTG TT-3′		
ETEC	*st*	F:5′-ATT TTT CTT TCT GTA TTG TCT T-3′	190	
		R:5′-CAC CCG GTA CAA GCA GGA TT-3′		
EIEC	*ial*	F:5′-GGT ATG ATG ATG ATG AGT CCA -3′	650	
		R:5′-GGAGGCCAACAATTATTTCC-3′		
EPEC	*eaeA*	F:5′-GAC CCG GCA CAA GCA TAA GC-3′	384	[58]
		R:5′-CCA CCT GCA GCA ACA AGA GG-3′		
STEC	*sxt2*	F:5′-GGC ACTGTCTGAAACTGCTCC-3′	255	
		R:5′-TCG CCA GTT ATC TGA CAT TCT G-3′		
EAEC	*aap*	F:5′-CTTGGGTATCAGCCTGAATG-3′	310	
		R:5′-AACCCATTC GGT TAG AGC AC- 3′		
DAEC	*afa*	F: 5'-GGCTTTTCTGCTGAACTGG-3'	809	
		R: 5'-CGGTCTCATAATCATGTCC-3'		
UPEC	*hlyA*	F:5′-AACAAGGATAAGCACTGTTCT -3′	1177	[22]
		R:5′ -ACCATATAAGCGGTCATTCCC- 3′		
UPEC	*cnf1*	F:5′-AAGATGGAGTTTCCTATGCAGGAG-3′	498	
		R:5′ -CATTCAGAGTCCTGCCCTCATTATT-3′		
UPEC	*vat*	F:5′-AGAGACGAGACTGTATTTGC-3′	289	
		R:5′-GTCAGGTCAGTAACGAGCAC- 3′		

Table S3. Primers sequences of the AR-genes.

Antibiotic Resistant	Locus	Primers	Amplicon (bp)	Reference
Tetracycline	*tetA*	F:5´-GCTACATCCTGCTTGCCTTC-3´<br>R:5´-CATAGATCGCCGTGAAGAGG- 3´	210	[59]
Sulfonamides	*sul1*	F:5´-GTGACGGTGTTCGGCATTCT-3´<br>R:5´-TTTACAGGAAGGCCAACGGT-3´	668	[60]
Chloramphenicol	*catA1*	F:5´-AGTTGCTCAATGTACCTATAACC-3´<br>R:5´-TTGTAATTCATTAAGCATTCTGCC- 3´	547	[61]
Beta lactams	*bla-tem*	F:5´-ACCAATGCTTAATCAGTGAG-3´<br>R:5´-GCGGAACCCCTATTTG-3´	963	[62]
Aminoglycosides	*strA*	F:5´-CTTGGTGATAACGGCAATTC-3´<br>R:5´-CCAATCGCAGATAGAAGGC- 3´	548	[63]
Polymyxin	*CLR5*	F:5´-CGGTCAGTCCGTTTGTTC- 3´<br>R:5´-CTTGGTCGGTCTGTA GGG - 3´	305	[64]
Carbapenem	*oxa48*	F:5´-GCTTGATCGCCCTCGATT-3´<br>R:5´-GATTTGCTCCGTGGCCGAAA-3´	281	[65]
Florfenicol	*floR*	F:5´ ACGTTTATGCCAACCGTCCT 3´<br>R: 5´CATTACAAGCGCGACAGTGG 3´	398	[66]
Quinolones	*qnrS*	F: 5-GCAAGTTCATTGAACAGGGT-3<br>F: 5-TCTAAACCGTCGAGTTCGGCG-3	428u	[67]

Table S4. Amplification programs used for each primer included in the study.

Gene	Amplification program	Reference
lt	50°C (2min, 1 cycle); 95°C (5min, 1 cycle); 95°C, 55, and 72°C (45seg each temperature, 40 cycles); and final extension (10 min, 72°C)	[57]
st	50°C (2min, 1 cycle); 95°C (5min, 1 cycle); 95°C, 55, and 72°C (45seg each temperature, 40 cycles); and final extension (10 min, 72°C)	[57]
eaeA	50°C (2min, 1 cycle); 95°C (5min, 1 cycle); 95°C, 55, and 72°C (45seg each temperature, 40 cycles); and final extension (10 min, 72°C)	[57]
sxt2	50°C (2min, 1 cycle); 95°C (5min, 1 cycle); 95°C, 55, and 72°C (45seg each temperature, 40 cycles); and final extension (10 min, 72°C)	[57]
ial	94°C/4min (1 cycle); 94°C/1min, 55°C/ 1min, 72°C/1min (35 cycles); final extension step (72°C/5min)	[67]
aap	50°C (2min, 1 cycle); 95°C (5min, 1 cycle); 95°C, 55, and 72°C (45seg each temperature, 40 cycles); and final extension (10 min, 72°C)	[68]
afa	50°C (2min, 1 cycle); 95°C (5min, 1 cycle); 95°C, 55, and 72°C (45seg each temperature, 40 cycles); and final extension (10 min, 72°C)	[68]
hlyA	94°C/3min (1 cycle); 94°C/1min, 60°C/30seg, and 72°C/3 min (30 cycles); and final extension step (72°C/7min)	[69]
cnf1	94°C/3min (1 cycle); 94°C/1min, 60°C/30seg, and 72°C/3 min (30 cycles); and final extension step (72°C/7min)	[69]
vat	95°C/5min (1 cycle); 94°C/30seg, 55°C/30seg, and 68°C/3 min (30 cycles); and final extension step (72°C/10min)	[70]
tetA	95°C/5min (1 cycle); 95/1min, 54°C/1min, 72°C/1min (30 cycles); final extension step (72°C/7min)	[59]
sul1	95°C/5min (1 cycle); 94/30 seg, 65°C/1min, 72°C/1.5min (30 cycles); final extension step (72°C/10min)	[60]
strA	95°C/5min (1 cycle); 95/1min, 54°C/1min, 72°C/1min (40 cycles); final extension step (72°C/7min)	[63]
catA1	94°C/5min (1 cycle); 94/30 seg, 50°C/30seg, 72°C/1.5min (30 cycles); final extension step (72°C/7min)	[71]
blatem	98°C/3min (1 cycle); 98/30 seg, 55°C/30seg, 72°C/30seg (40 cycles); final extension step (72°C/5min)	[62]
CLR5	50°C (2min, 1 cycle); 95°C (10min, 1 cycle); 95°C/15 seg, 60°C/1min (40 cycles)	[64]
oxa48	94°C/10min (1 cycle); 94/40 seg, 60°C/40seg, 72°C/1min (30 cycles); final extension step (72°C/7min)	[65]
floR	94°C/3min (1 cycle); 94/40 seg, 58°C/40seg, 75°C/40seg (35 cycles); final extension step (75°C/7min)	[72]
qnrS	95°C/10min (1 cycle); 95°C/1min, 55°C/ 1min, 72°C/1min (35 cycles); final extension step (72°C/10min)	[73]

Figure S4. Antibiotic resistance mechanisms detected among the *E. coli* isolated from food. **(1)** Tetracycline resistance mediated by the efflux pump with an antiport system. **(2)** Sulfonamide resistance is carried out by the modified dihydropteroate synthase enzyme. **(3)** Aminoglycoside resistance is allowed by the activity of the aminoglycoside-phosphotransferase enzyme that modifies the antibiotic structure. **(4)** Quinolone resistance mechanisms in which the QnrS protein decreases the activity of these antibiotics. **(5)** Chloramphenicol resistance is mediated by the acetylation of the drug structure by the chloramphenicol-acetyltransferase. Created in https://BioRender.com.

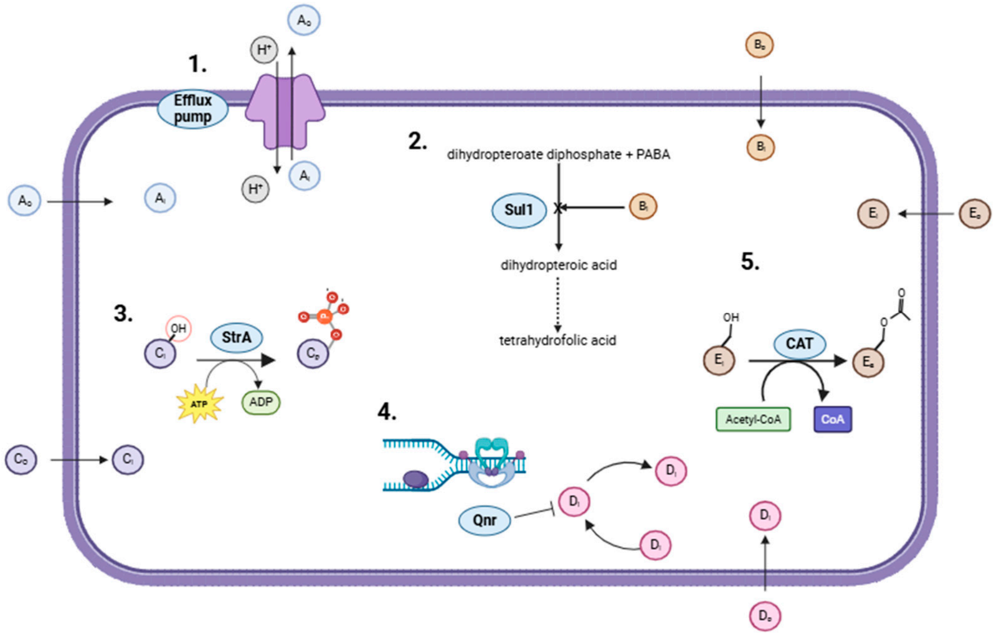

Supplement: Supplementary file 1 [file antibiotics-14-00406-s001.zip › antibiotics-3538990-supplementary.pdf]
